# Ellagic Acid Ameliorates Diabetic Cardiomyopathy by Inhibiting Ferroptosis Through the Modulation of the SIRT1/p53 Pathway in Streptozotocin-Induced Diabetic Rats

**DOI:** 10.5812/ijpr-166600

**Published:** 2025-12-02

**Authors:** Qingmei Wang, Xuanguo Zhang, Li Xi

**Affiliations:** 1 Department of Health Management Center, Shandong Provincial Third Hospital, Shandong University, Jinan 250031, China; 2 Department of Intensive Care Unit, Shaanxi Traditional Chinese Medicine Hospital, Xi'an, 710003, China; 3 Editorial Department of Shaanxi Journal of Traditional Chinese Medicine, Shaanxi Academy of Traditional Chinese Medicine, Xi'an, 710003, China

**Keywords:** Oxidative Stress, Hypoglycemic Agents, Phytochemicals, Lipid Metabolism, Cell Death

## Abstract

**Background:**

Diabetic cardiomyopathy (DCM) involves ferroptosis, an iron-dependent cell death pathway. Ellagic acid (EA), a natural antioxidant flavonoid, may offer therapeutic potential; however, its mechanisms in DCM remain unexplored.

**Objectives:**

This study investigated the cardioprotective effects of EA in experimental DCM, focusing on its capacity to mitigate ferroptosis via the sirtuin 1 (SIRT1)/p53 pathway.

**Methods:**

The EA (25, 50, or 100 mg/kg/day) was orally administered to streptozotocin (STZ)-induced diabetic rats for 60 days. We assessed cardiac function, histology, metabolic parameters, oxidative stress, inflammation, and key markers of ferroptosis and the SIRT1/p53 axis. Data were analyzed by one-way analysis of variance (ANOVA) with Tukey's post-hoc test.

**Results:**

The EA treatment dose-dependently attenuated cardiac hypertrophy, myocardial injury, and metabolic dysregulation, with maximal benefits at 100 mg/kg. It also reduced oxidative stress and inflammation. Crucially, EA inhibited ferroptosis, as evidenced by reduced iron overload and upregulation of solute carrier family 7 member 11 (SLC7A11) and glutathione peroxidase 4 (GPX4). These benefits were associated with the upregulation of SIRT1 and downregulation of p53 in cardiac tissue.

**Conclusions:**

The EA mitigates DCM by suppressing ferroptosis, potentially through modulation of the SIRT1/p53 pathway, thereby improving cardiac function and metabolic homeostasis. However, as this study utilized an STZ-induced model of type 1 diabetes, further research is warranted to confirm its efficacy in type 2 diabetic contexts.

## 1. Background

Diabetes mellitus is a pervasive global health challenge, with prevalence projected to rise from 463 million cases in 2019 to 700 million by 2045 (1, 2). A major complication is diabetic cardiomyopathy (DCM), characterized by ventricular hypertrophy and systolic/diastolic dysfunction, which remains a leading cause of mortality despite current therapies (3, 4). The pathophysiology of DCM involves multiple factors, including the accumulation of cytotoxic advanced glycation end-products (AGEs) and lipid intermediates that drive reactive oxygen species (ROS) overproduction (5, 6), alongside dysregulated cell death pathways. Importantly, ferroptosis, an iron-dependent form of regulated cell death driven by lipid peroxidation (7), has emerged as a critical mediator of myocardial damage in diabetes (8). Iron overload promotes the peroxidation of membrane phospholipids, disrupting redox homeostasis and directly triggering cardiomyocyte death.

Current DCM management, focused on glycemic control (CON), inadequately addresses heart failure risk, highlighting the need for targeted therapies (9). Natural polyphenols represent promising candidates for DCM (10). For instance, ellagic acid (EA), a bioactive flavonoid abundant in pomegranate and berries, has demonstrated direct cardioprotective and anti-diabetic properties in preclinical models. Studies have shown that EA attenuates cardiac hypertrophy and fibrosis in hypertensive rats (11) and improves glycemic CON in streptozotocin (STZ)-induced diabetic models (12). Furthermore, emerging evidence suggests that EA can inhibit ferroptosis in neuronal and hepatic injury models (13, 14). However, its specific role in modulating cardiac ferroptosis within the context of DCM remains entirely uncharacterized.

## 2. Objectives

Given the multifactorial pathogenesis of DCM, this study investigates the cardioprotective potential of EA. The present study specifically evaluates the hypothesis that EA mitigates DCM by inhibiting ferroptosis and focuses on the modulation of the sirtuin 1 (SIRT1)/p53 axis, a key pathway governing iron-dependent cell death. The findings may address a significant knowledge gap in diabetic cardiovascular therapeutics.

## 3. Methods

### 3.1. Chemicals

The EA dihydrate (CAS 476-66-4; MW 302.19 g/mol; purity ≥ 95%) was sourced from Sigma-Aldrich (St. Louis, MO), along with STZ and protease inhibitors. Beyotime Biotechnology (Shanghai, China) provided the BCA Protein Assay Kits and Radioimmunoprecipitation Assay (RIPA) Lysis Buffer. Abcam (Cambridge, UK) supplied enzyme-linked immunosorbent assay (ELISA) kits for inflammatory cytokines [interleukin-1 beta (IL-1β), interleukin-6 (IL-6), interleukin-4 (IL-4), interleukin-10 (IL-10)] and oxidative stress markers [superoxide dismutase (SOD), catalase (CAT), glutathione (GSH), malondialdehyde (MDA)]. Anesthesia reagents (ketamine 10%; xylazine 2%) were procured from Bremer Pharma (Warburg, Germany) and Alfasan (Woerden, Netherlands), respectively.

### 3.2. Experimental Animals and Diabetic Model

Forty male albino Wistar rats (8 weeks; 200 - 250 g) were maintained under controlled conditions (22 ± 3°C; 12-hour light/dark cycle; and 50 ± 5% humidity) with ad libitum access to a standard laboratory rodent diet (Teklad Global 18% Protein Rodent Diet) and water. No supportive care (e.g., insulin) or analgesics were administered during the study to avoid confounding the diabetic state and the investigated parameters. Diabetes was induced via intraperitoneal injection of STZ (45 mg/kg in pH 4.5 citrate buffer) after 12-hour fasting. The CON animals received citrate buffer alone. Diabetic status was confirmed by fasting blood glucose > 250 mg/dL on three consecutive measurements 7 days post-injection. Normoglycemic controls maintained blood glucose levels of 80 - 100 mg/dL. The induction of diabetes in the experimental cohort was accomplished through the application of well-established protocols (15). At the end of the experimental period, animals were euthanized by an overdose of the anesthetic cocktail (ketamine/xylazine) followed by exsanguination, in strict accordance with the approved ethical guidelines.

### 3.3. Treatment Groups and Protocol

Animals were stratified into five cohorts (n = 8/group).

1. The CON: Non-diabetic+saline gavage.

2. The DCM: Diabetic+saline gavage.

3. EA25: Diabetic + 25 mg/kg/day EA.

4. EA50: Diabetic + 50 mg/kg/day EA.

5. EA100: Diabetic + 100 mg/kg/day EA.

Sample size (n = 8 per group) was determined based on previous studies in similar DCM models (16) and our pilot data, which indicated that this provides adequate power (≥ 80%) to detect significant differences in key parameters including cardiac hypertrophy indices, metabolic parameters, and molecular markers with α = 0.05. The doses of EA (25, 50, and 100 mg/kg/day) were selected based on previous preclinical studies demonstrating efficacy and safety in rodent models of diabetes and cardiovascular complications (11, 17). Treatments were initiated 14 days after the confirmation of diabetes and were administered daily via oral gavage for a subsequent period of 60 days. Thus, the total study duration from STZ injection to the endpoint was 74 days. All oral gavage procedures were performed at a fixed time each morning (between 9:00 and 10:00 AM) to maintain consistent pharmacodynamic conditions. All forty animals completed the entire study protocol with no unexpected mortality, and all were included in the final analyses. Body weight was recorded biweekly. Primary outcomes included cardiac function (hemodynamic parameters, cardiac injury biomarkers) and cardiac histology. Secondary outcomes encompassed metabolic parameters (glucose tolerance, lipid profiles), oxidative stress markers, inflammatory cytokines, and molecular markers of ferroptosis and the SIRT1/p53 pathway.

### 3.4. The Analysis of Physical and Cardiac Indices

Subsequent to euthanasia, specimens were carefully separated to ensure precise weight measurements. In order to assess cardiac hypertrophy progression, the cardiac weight-to-body weight ratio (HW/BW ratio) was calculated using the following formula: Cardiac weight-to-body weight (CW/BW) ratio = Cardiac weight/Body weight. Tissues and chemicals were weighed using a Sartorius CPA224S analytical balance. Furthermore, blood pressure measurements were taken upon completion of the experimental procedure utilizing tail-cuff plethysmography on awake animals that had been preheated in a temperature-regulated restraint device (XBP1000, Kent Scientific) for 10 minutes (18). Diastolic blood pressure (DBP) and systolic blood pressure (SBP) were determined by averaging 3 - 5 measurements taken during three separate sessions. Additionally, levels of the cardiac biomarkers troponin T and creatine kinase-myocardial band (CK-MB) were measured.

### 3.5. The Measurement of Glycemic and Lipid Profiles

A standardized protocol was followed to conduct glucose tolerance tests and insulin tolerance tests (GTT and ITT). Additionally, the plasma biochemical parameters, which encompass lipid profiles [triglycerides (TG), high-density lipoprotein (HDL), low-density lipoprotein (LDL), and total cholesterol (TC)] along with fasting blood sugar (FBS) levels, were analyzed utilizing MyBioSource reagents, adhering strictly to the manufacturer's instructions.

### 3.6. Histological Analysis of Cardiac Tissues

Cardiac tissue specimens were fixed in 10% buffered formalin for 24 hours. Subsequently, tissues were dehydrated using a graded ethanol series. Following dehydration, tissues were embedded in paraffin wax. Sections of 5 µm thickness were prepared from the paraffin blocks using a microtome (Viabrembo, Milan, Italy). The sections were then dewaxed in xylene, rehydrated through a descending ethanol gradient, and finally rinsed in sterile distilled water. Hematoxylin and eosin (H&E) staining was performed according to standard protocols (11). Stained sections were examined and imaged using light microscopy (Nikon, Tokyo, Japan).

### 3.7. Enzyme-Linked Immunosorbent Assay

The cardiac tissues, each weighing approximately half a gram, were homogenized with a RIPA buffer that included protease inhibitors. Consequently, inflammatory cytokines such as IL-1β, IL-6, IL-4, and IL-10 were measured in the cardiac tissue homogenates using ELISA kits provided by Abcam. The analysis was executed in accordance with the manufacturer's instructions, and a BioTek Synergy H1 microplate reader was used for absorbance measurement. Following this, the focus shifted to the detection of four significant proteins: The SIRT1, p53, glutathione peroxidase 4 (GPX4), and solute carrier family 7 member 11 (SLC7A11). Moreover, tissue levels of iron were measured in the cardiac homogenates, with strict compliance with the protocols recommended by the manufacturer.

### 3.8. Analysis of Oxidative Stress

Homogenized cardiac tissues were evaluated for oxidative stress by measuring the activity of SOD, glutathione reductase (GR), and CAT, along with reduced GSH and MDA concentrations. Absorbance was measured using a BioTek Synergy H1 microplate reader. Appropriate commercial kits were utilized in strict adherence to the manufacturer's protocols to maintain precision.

### 3.9. The Analysis of Gene Expression

The PureLink RNA Mini Kit (catalog number: 12183018A, Thermo Fisher Scientific, USA) was used to extract total RNA following the manufacturer's protocol. The Biotek Nanodrop system measured RNA quality and quantity. For complementary DNA (cDNA) synthesis, the High-Capacity cDNA Reverse Transcription Kit (catalog number: 4368814, Thermo Fisher Scientific, USA) was employed. Subsequent quantitative polymerase chain reaction (qPCR) analysis used the StepOne Real-time PCR System (Applied Biosystems, USA) and Maxima SYBR Green qPCR Master Mix (catalog number: K0253, Thermo Fisher Scientific, USA). Glyceraldehyde-3-phosphate dehydrogenase (GAPDH) served as the normalization control for gene expression levels. Fold changes in expression were calculated using the 2^-ΔΔCT^ method. All primers used in this study are listed in Table 1. 

**Table 1.** Primer Sequences

Primers	Sequence
**GAPDH**	
Forward	5′-GGTGGACCTCATGGCCTACAT-3′
Reverse	5′-GCCTCTCTCTTGCTCTCAGTATCCT-3′
**p53**	
Forward	5′-TTCCCTCAATAAGCTGTTCTG CC-3′
Reverse	5′-TGCTCAAGTTCCACTAGCTGG-3′
**SIRT1**	
Forward	5′-GAGTAGTCTATAGGTTACGTGG-3′
Reverse	5′-AAATATGAAGAGGTGTTGGTGG-3′
**GPX4**	
Forward	5′-ACGCCAAAGTCCTAGGAAGC-3′
Reverse	5′-CTGCGAATTCGTGCATGGAG-3′
**SLC7A11**	
Forward	5′-GACAGTGTGTGCATCCCCTT-3′
Reverse	5′-GCATGCATTTCTTGCACAGTTC-3′

Abbreviations: GAPDH, glyceraldehyde-3-phosphate dehydrogenase; SIRT1, sirtuin 1; GPX4, glutathione peroxidase 4; SLC7A11, solute carrier family 7 member 11.

### 3.10. Statistical Analysis

Data are presented as mean ± SD. Intergroup differences were assessed by one-way analysis of variance (ANOVA; SPSS v24.0) with Tukey's post-hoc test. Time-course data were analyzed via two-way ANOVA. The significance threshold was set at P < 0.05. Figures were generated with GraphPad Prism v8.

## 4. Results

### 4.1. Ellagic Acid Alleviated Physical and Metabolic Markers in Diabetic Cardiomyopathy Animals

**Figure 1.**
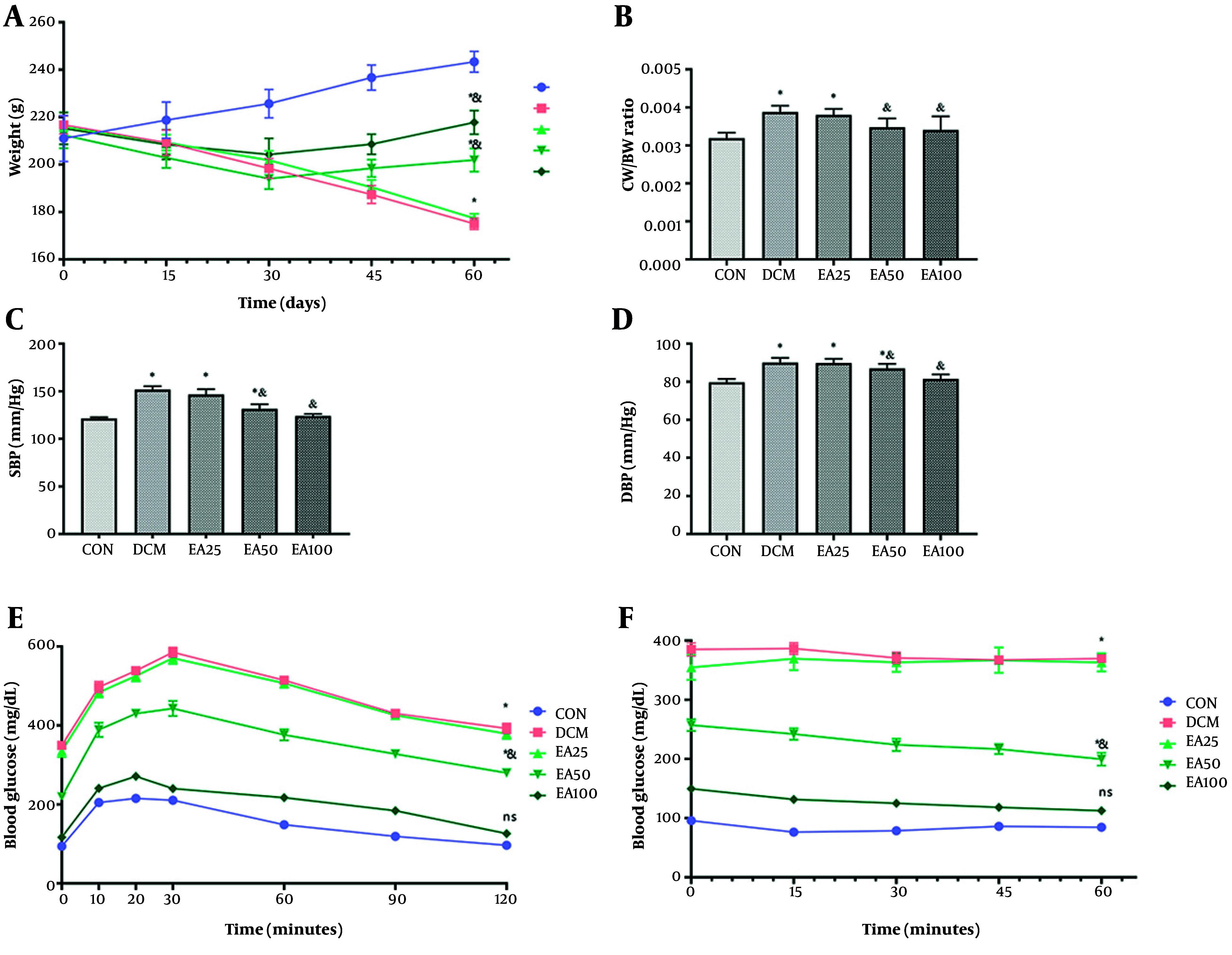
Effects of ellagic acid (EA) on physical, biochemical, and cardiac markers in experimental groups over 60 days: A, mean body weight; B, cardiac weight-to-body weight (CW/BW) ratio; C, systolic blood pressure (SBP); D, diastolic blood pressure (DBP); E, GTT; and F, ITT of rats in control (CON), diabetic cardiomyopathy (DCM), and EA-treated diabetic groups (EA25, EA50, EA100). Different symbols (* and &) indicate significant differences (P < 0.05) while shared letters denote no significant difference. Groups that do not share a common letter are significantly different.

Treatment with EA (100 mg/kg/day) significantly attenuated DCM-associated pathophysiology. Diabetic rats exhibited reduced body weight versus CONs (P = 0.009), with EA100 partially restoring mass (P < 0.001). Cardiac hypertrophy, assessed by CW/BW ratio, showed complete normalization in EA100 versus DCM (P = 0.002) and CON (P > 0.355). Hemodynamic parameters revealed EA100-mediated reduction of systolic and diastolic pressures compared to DCM (P < 0.001, Figure 1). The analysis of tolerance to glucose and insulin showed that all studied groups had a significant increment in comparison with normoglycemic rats (P < 0.05). However, the EA100 group represented a significant difference with DCM animals as well (P < 0.01). Metabolic profiling demonstrated EA100's efficacy in glucose regulation (103.46 ± 10.47 mg/dL vs. DCM 326.28 ± 7.99 mg/dL, P < 0.01) and lipid homeostasis, including HDL restoration: 42.80 ± 2.57 mg/dL (DCM: 28.12 ± 1.50), LDL reduction: 51.40 ± 5.97 mg/dL (DCM: 91.88 ± 2.86), TG normalization: 68.06 ± 5.82 mg/dL (DCM: 158.39 ± 8.13), and TC normalization: 72.88 ± 8.43 mg/dL (DCM: 141.51 ± 11.08). Cardiac injury biomarkers troponin T and CK-MB in EA100 approached CON levels versus DCM (P < 0.05, Table 2). 

**Table 2.** Effects of Ellagic Acid on Metabolic Parameters and Cardiac Injury Markers in Experimental Groups ^a, b^

Variables	CON	DCM	EA25	EA50	EA100
**Glucose**	90.26 ± 1.68	326.28 ± 7.99 ^c^	294.14 ± 26.02 ^d^	173.82 ± 15.06 ^d^	103.46 ± 10.47 ^e^
**HDL**	43.80 ± 2.04	28.12 ± 1.50 ^c^	29.15 ± 1.72 ^c^	37.11 ± 2.54 ^d^	42.80 ± 2.57 ^e^
**LDL**	38.34 ± 2.16	91.88 ± 2.86 ^c^	91.92 ± 2.69 ^c^	69.96 ± 8.88 ^d^	51.40 ± 5.97 ^d^
**TG**	50.52 ± 2.67	158.39 ± 8.13 ^c^	157.04 ± 6.47 ^c^	97.84 ± 6.84 ^d^	68.06 ± 5.82 ^d^
**TC**	79.34 ± 3.91	141.51 ± 11.08 ^c^	122.51 ± 11.09 ^c^	77.83 ± 7.08 ^e^	72.88 ± 8.43 ^e^
**Troponin T**	66.16 ± 0.55	191.61 ± 5.61 ^c^	190.11 ± 2.88 ^c^	106.99 ± 6.43 ^d^	68.92 ± 3.86 ^e^
**CK-MB**	181.08 ± 13.30	447.50 ± 20.84 ^c^	441.51 ± 21.17 ^c^	312.74 ± 32.91 ^d^	210.43 ± 17.85 ^e^

Abbreviations: CON, control; DCM, diabetic cardiomyopathy; HDL, high-density lipoprotein; LDL, low-density lipoprotein; TG, triglycerides; TC, total cholesterol; CK-MB, creatine kinase-myocardial band.

^a^ Values are expressed as mean ± SD.

^b^ Fasting blood glucose (mg/dL), lipid profiles (HDL, LDL, TG, and TC all in mg/dL), and cardiac biomarkers (troponin T in ng/mL and CK-MB in U/L) in CON, DCM, and ellagic acid (EA)-treated diabetic rats (EA25, EA50, EA100).

^c^ Significant difference compared to CON.

^d^ Significant difference compared to both CON and DCM (P-value < 0.05).

^e^ Significant difference compared to DCM.

### 4.2. Effect of Ellagic Acid on Cardiac Histomorphology in Diabetic Cardiomyopathy Rats

**Figure 2.**
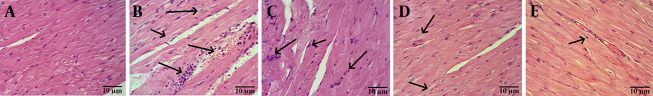
Histopathological analysis of cardiac tissues: A, control (CON): Normal architecture with striated cardiomyocytes, central nuclei, and linear fiber arrays; B, diabetic cardiomyopathy (DCM): Arrows indicate loss of striation and disrupted anastomoses; C, EA25: Similar pathology to DCM; D, EA50: Arrows indicate partial restoration of striations; E, EA100: Arrows indicate near-normal histoarchitecture with restored striations (Abbreviation: H&E, hematoxylin and eosin staining; scale bar: 10 μm; original magnification 400X).

Histomorphological analysis revealed that cardiac tissue from CON animals exhibited preserved architecture, characterized by striated cardiomyocytes with centrally located nuclei and anastomosing/branched fibers organized in linear arrays. Conversely, cardiac tissue from DCM rats and rats treated with EA25 and EA50 displayed a loss of striation and anastomoses, alongside the presence of intercalated discs. Notably, histopathological assessment indicated that EA100 treatment resulted in amelioration of cardiac tissue histoarchitecture compared to the untreated DCM group (Figure 2). 

### 4.3. Ellagic Acid Attenuated Cardiac Inflammation and Oxidative Stress

**Figure 3.**
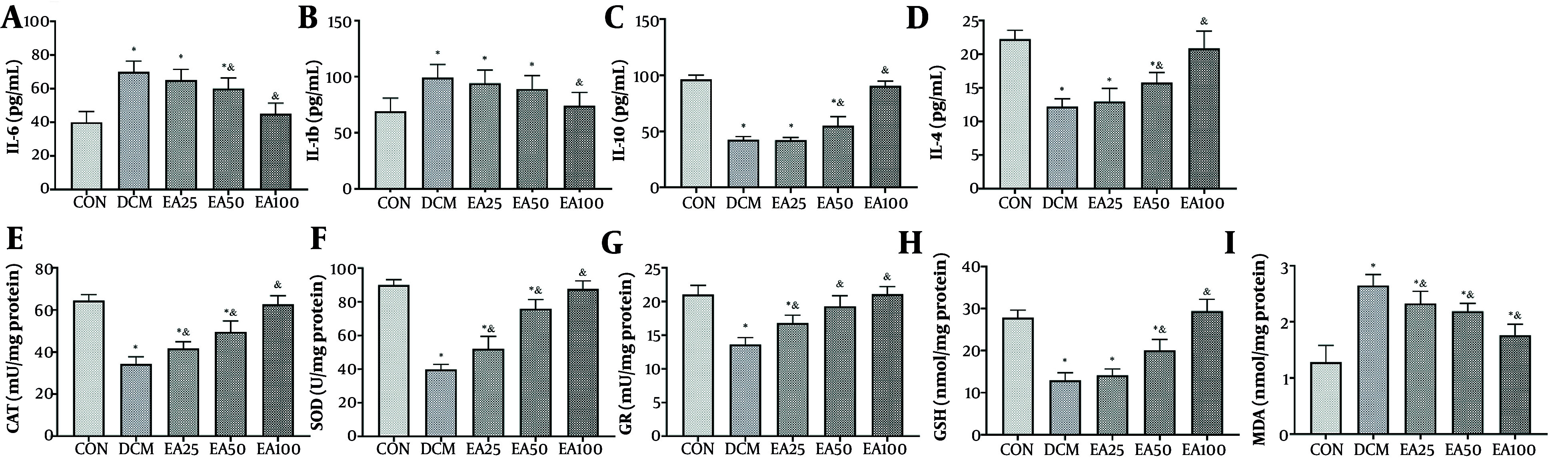
Effects of ellagic acid (EA) on inflammation and oxidative stress markers in cardiac tissue: The levels of inflammatory markers including A, interleukin-6 (IL-6); B, interleukin-1 beta (IL-1β); C, interleukin-10 (IL-10); and D, interleukin-4 (IL-4, pg/mL) along with oxidative stress markers including E, catalase (CAT) activity (mU/mg protein); F, superoxide dismutase (SOD) activity (U/mg protein); G, glutathione reductase (GR, mU/mg protein); H, glutathione (GSH) levels (nmol/mg protein); and I, malondialdehyde (MDA) levels (nmol/mg protein) are depicted. Different symbols (* and &) indicate significant differences (P < 0.05) while shared letters denote no significant difference. Groups that do not share a common letter are significantly different [values are expressed as mean ± SD).

Administration of EA (100 mg/kg/day) significantly modulated inflammatory mediators in cardiac tissue. Pro-inflammatory cytokines IL-6 (P < 0.001) and IL-1β (P = 0.002) were reduced significantly versus DCM controls. Concurrently, anti-inflammatory IL-10 and IL-4 increased 2-fold and 2.1-fold compared to DCM (P < 0.001). The EA also restored redox homeostasis, enhancing antioxidant enzyme activities, such as the activity of CAT, SOD, and GR, and levels of reduced GSH increased more than 40% (P < 0.001) relative to DCM, while reducing the lipid peroxidation marker MDA by over 60% (P < 0.001, Figure 3). 

### 4.4. Ellagic Acid Modified Ferroptotic Markers

**Figure 4.**
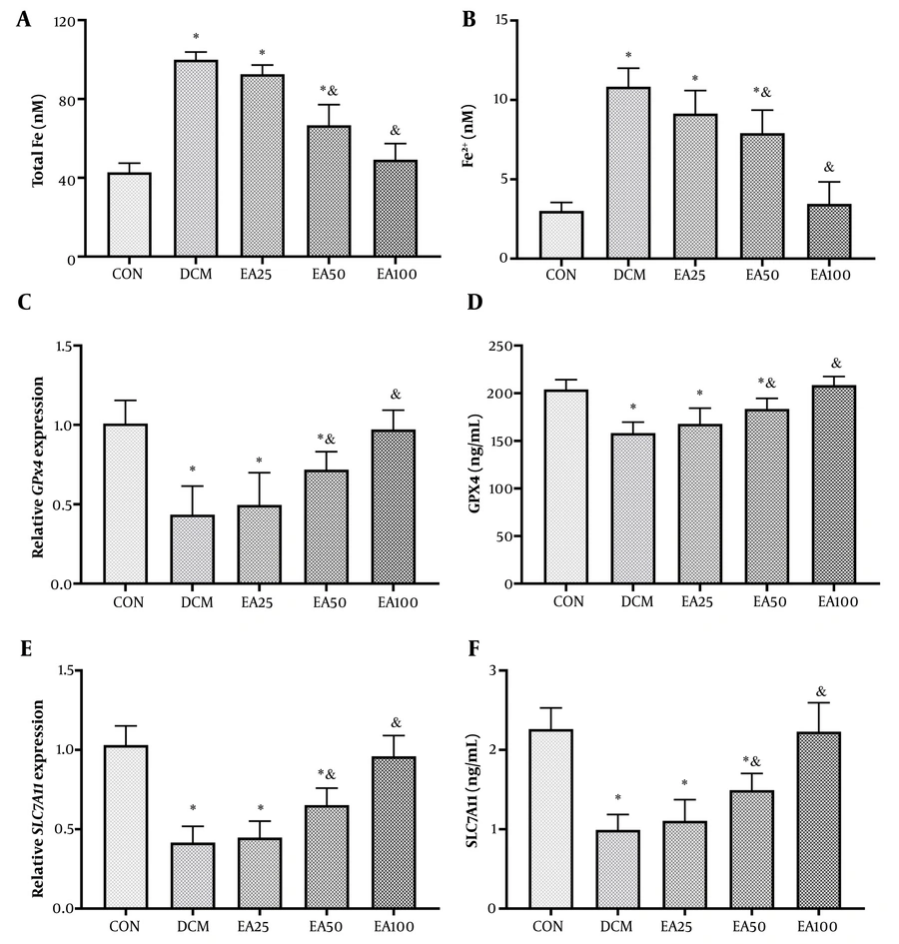
Ellagic acid (EA) modulates ferroptosis markers in diabetic cardiomyopathy (DCM) rats: A, serum total iron (nM); B, Fe^2+^ (nM); C, glutathione peroxidase 4 (GPX4) mRNA [fold change vs. control (CON)]; D, GPX4 protein (ng/mL); E, solute carrier family 7 member 11 (SLC7A11) mRNA (fold change vs. CON); and F, SLC7A11 protein (ng/mL). Different symbols (* and &) indicate significant differences (P < 0.05) while shared letters denote no significant difference. Groups that do not share a common letter are significantly different.

The EA treatment (100 mg/kg/day) effectively reversed ferroptosis indicators. Serum analysis revealed a 42% reduction in total iron (P < 0.001) and a 51% decrease in Fe^2+^ (P < 0.001) compared to DCM. Molecular assessments demonstrated coordinated upregulation of ferroptosis defense mechanisms, as SLC7A11 gene expression increased 3.8-fold (P < 0.001) with a corresponding protein elevation (P < 0.001). Similarly, GPX4 expression rose 4.2-fold (P < 0.001), with protein levels reaching 36.5 ± 3.1 ng/mL (vs. DCM 15.9 ± 1.8 ng/mL; P < 0.001, Figure 4). 

### 4.5. Ellagic Acid Modulated Upstream Ferroptosis Regulators in Cardiac Tissue of Diabetic Cardiomyopathy Animals

**Figure 5.**
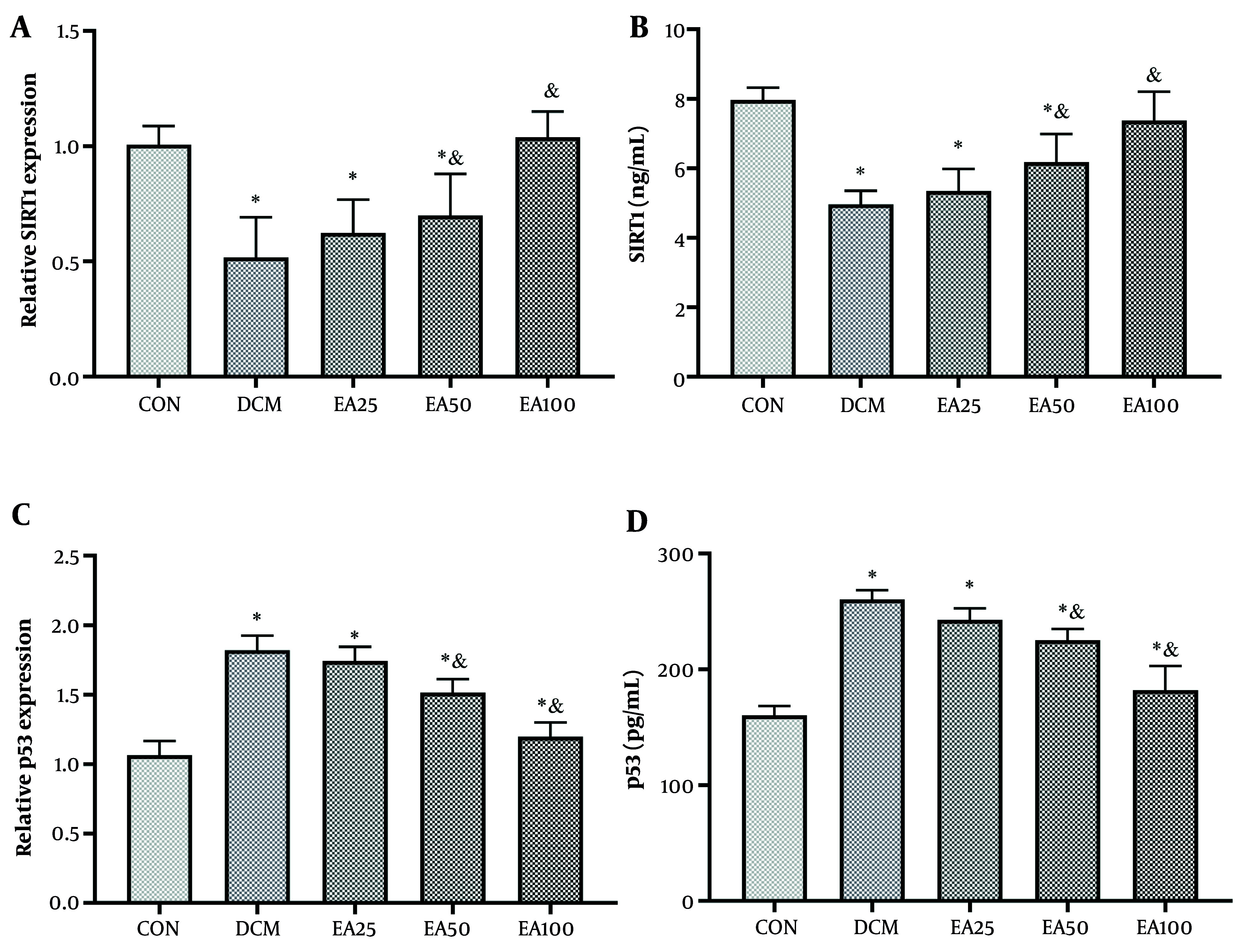
Ellagic acid (EA) regulates the sirtuin 1 (SIRT1)/p53 pathway in cardiac tissue: A, SIRT1 mRNA [fold change vs. control (CON)]; B, SIRT1 protein (ng/mL); C, p53 mRNA (fold change vs. CON); and D, p53 protein (ng/mL). Different symbols (* and &) indicate significant differences (P < 0.05) while shared letters denote no significant difference. Groups that do not share a common letter are significantly different.

The SIRT1 and p53 have been recognized in multiple studies as key upstream modulators of ferroptosis, making them promising treatment options for addressing disease resulting from ferroptosis-dependent cell death (19, 20). The current investigation revealed that in the cardiac tissue of DCM rats, the expression levels of SIRT1- and p53-encoding genes, along with the concentrations of SIRT1 and p53 proteins, exhibited a significant decline and an increase, respectively, compared to CON animals (P < 0.001). No significant difference was found between DCM rats treated with 25 mg/kg/day EA and the DCM group (P > 0.05). However, EA50 animals showed a significant difference from both DCM and CON animals regarding SIRT1 gene expression and protein levels (P < 0.05). Conversely, the EA100 group demonstrated a notable improvement in the expression of the SIRT1 coding gene and the level of SIRT1 protein when compared to DCM rats (P < 0.001). Similarly, EA25 did not represent a significant difference compared to DCM regarding p53 gene expression and protein levels (P > 0.05); EA50 and EA100 represented a significant difference from both CON and DCM animals (P < 0.05, Figure 5). 

## 5. Discussion

The DCM is pathologically associated with cardiac hypertrophy and hypertension (21). The EA treatment (100 mg/kg/day) effectively reduced FBS and normalized lipid profiles. These cardiometabolic improvements are consistent with the established capacity of phytochemicals such as EA to enhance endocrine and metabolic homeostasis via antioxidant and anti-inflammatory mechanisms (22-25). Accordingly, EA administration significantly reduced cardiac tissue levels of pro-inflammatory cytokines (IL-6 and IL-1β) and elevated anti-inflammatory cytokines (IL-10 and IL-4) in DCM rats. Furthermore, EA treatment enhanced the activity of key antioxidant enzymes (SOD, GR, and CAT) and increased reduced GSH levels in cardiac tissue, while reducing lipid peroxidation (measured by MDA). Oxidative stress, characterized by disrupted glucolipid metabolism, inflammation, and an imbalance between free radical generation and antioxidant defenses, is a well-established pathophysiological mechanism in DCM initiation and progression (5, 26). Consequently, targeting oxidative stress signaling pathways remains a promising therapeutic approach for DCM.

The DCM is a complex pathology; hence the molecular mechanisms driving DCM progression remain incompletely elucidated. The DCM typically advances through cardiac fibrosis to heart failure, a process strongly associated with cardiomyocyte death (27, 28). This study demonstrated that EA administration significantly ameliorated serum iron overload in diabetic rats, reducing both total iron and Fe^2+^ levels. Iron overload directly promotes tissue damage through increased ROS generation, elevated lipid peroxidation, and GSH depletion (29). The observed attenuation of ferroptosis by EA is consistent with its antioxidant properties, aligning with established roles of antioxidants in inhibiting ferroptosis. Key molecular markers of ferroptosis include the downregulation of SLC7A11 (a cystine/glutamate antiporter essential for GSH synthesis) and GPX4 (a GSH-dependent enzyme that reduces lipid hydroperoxides to mitigate oxidative damage) (8). Crucially, in diabetic rats, EA treatment upregulated the expression of both SLC7A11 and GPX4 genes and increased corresponding protein levels in cardiac tissue. This concomitant upregulation of core ferroptosis defense components mechanistically supports EA-mediated inhibition of ferroptosis in DCM.

While reducing inflammation and oxidative stress can inhibit ferroptosis, identifying the upstream regulators is critical. Therefore, this study investigated the SIRT1/p53 axis. The SIRT1 is a nicotinamide adenine dinucleotide (NAD^+^)-dependent deacetylase that regulates inflammation, cell death, and mitochondrial function. It acts by deacetylating both histone and non-histone substrates (30). Deubiquitination-mediated stabilization of SIRT1 inhibits pro-apoptotic pathways, notably by suppressing the transcriptional activity of p53 (31, 32). Reduced SIRT1 activity correlates with p53 overexpression, a key driver of chronic cardiovascular pathologies including DCM (33, 34). Significantly, p53 is a primary ferroptosis inducer that transcriptionally represses SLC7A11, limiting cystine uptake and GSH synthesis, and downregulates GPX4. Consequently, the SIRT1/p53 axis is recognized as a principal regulator of ferroptosis and a promising therapeutic target, particularly for phytochemicals. In alignment with this paradigm, the present study demonstrates that EA administration (100 mg/kg/day) significantly upregulated SIRT1 expression while downregulating p53 in cardiac tissue. These findings indicate that EA restores SLC7A11 and GPX4 expression, thereby inhibiting ferroptosis in DCM, through modulation of the SIRT1/p53 signaling pathway. Importantly, the observed dose-dependent efficacy of EA, with the 100 mg/kg dose producing the most significant restoration of physiological and molecular parameters, provides strong internal validation of its therapeutic effect and argues against non-specific actions.

The present findings hold significant translational promise, suggesting EA as a possible adjunct therapy for DCM. However, key limitations warrant consideration. First, the exclusive use of a STZ-induced model of type 1 diabetes may not fully recapitulate type 2 DCM pathophysiology. Moreover, EA's well-documented poor oral bioavailability in humans presents a major pharmacological challenge. Achieving effective cardiac tissue concentrations may require pharmaceutical optimization through advanced delivery systems such as nano-formulations. Also, while our 60-day treatment showed no adverse effects, long-term safety and cardiac-specific outcomes beyond this period remain unvalidated.

Additionally, while our data demonstrate a strong association between EA treatment and modulation of the SIRT1/p53 pathway, we acknowledge that this evidence is correlative rather than definitively causal. Future studies using SIRT1 inhibitors or p53 activators would be needed to establish direct mechanistic causality. The present study assessed total p53 protein levels but did not evaluate its active form (e.g., phosphorylated p53), which would provide deeper insight into its functional state. The inclusion of a known ferroptosis inhibitor (e.g., ferrostatin-1) as a positive CON would have further strengthened present conclusions by providing a direct comparison for EA's anti-ferroptotic effects. In addition, detailed correlation analysis between inflammatory markers and ferroptosis indicators was not performed, which could have provided additional insight into their relationship. Moreover, ferroptosis was assessed primarily through systemic iron measurements and molecular markers. While we demonstrated a clear upregulation of the key ferroptosis defense proteins SLC7A11 and GPX4 in cardiac tissue, future studies would benefit from direct quantification of cardiac tissue iron content using techniques such as inductively coupled plasma mass spectrometry (ICP-MS) to provide an even more direct link. Furthermore, while present H&E staining demonstrated clear improvement in cardiac tissue architecture with EA treatment, the authors acknowledge that more specific histological techniques would strengthen our findings.

Future studies should prioritize validating these mechanisms in diabetic large mammals (e.g., pigs) with inherent metabolic similarities to humans, evaluating EA in combination with standard-of-care cardioprotective agents [e.g., sodium-glucose cotransporter 2 (SGLT2) inhibitors], and investigating dose-dependent effects on human cardiomyocytes using three-dimensional (3D) organoid models. Until such data are available, clinical translation should remain cautious, though our results robustly nominate EA as a novel ferroptosis-targeting candidate for DCM management.

### 5.1. Conclusions

In conclusion, this study demonstrates that EA administration, particularly at 100 mg/kg/day, ameliorates STZ-induced DCM in rats through multifaceted mechanisms. The EA significantly attenuated cardiac hypertrophy, hypertension, and histopathological damage while normalizing metabolic dysregulation (glucose intolerance, LDL, TG, and HDL) and reducing myocardial injury biomarkers (troponin T and CK-MB). Crucially, EA suppressed ferroptosis by reducing systemic iron overload (total Fe and Fe^2+^), upregulating key ferroptosis inhibitors (SLC7A11 and GPX4), and restoring redox balance (increased SOD, CAT, and GSH; decreased MDA). These effects were mediated via modulation of the SIRT1/p53 axis, wherein EA upregulated SIRT1 expression while suppressing p53 activation. Collectively, these findings establish EA as a promising phytotherapeutic agent against DCM, primarily through ferroptosis inhibition orchestrated by SIRT1/p53 pathway regulation, suggesting its potential role in clinical evaluations for diabetic cardiovascular complications.

## Data Availability

The data that support the findings of this study are available from the corresponding author upon reasonable request.
